# Epigenetic regulation of the neural transcriptome and alcohol interference during development

**DOI:** 10.3389/fgene.2014.00285

**Published:** 2014-08-26

**Authors:** Marisol Resendiz, Stephen Mason, Chiao-Ling Lo, Feng C. Zhou

**Affiliations:** ^1^Stark Neuroscience Research InstituteIndianapolis, IN, USA; ^2^Indiana Alcohol Research Center, Indiana University School of MedicineIndianapolis, IN, USA; ^3^Department of Anatomy and Cell Biology, Indiana University School of MedicineIndianapolis, IN, USA

**Keywords:** neuroepigenetics, neural stem cells, DNA methylation, histone modification, miRNA, epigenomics, neural developmental pathway, gene–environment interaction

## Abstract

Alcohol intoxicated cells broadly alter their metabolites – among them methyl and acetic acid can alter the DNA and histone epigenetic codes. Together with the promiscuous effect of alcohol on enzyme activities (including DNA methyltransferases) and the downstream effect on microRNA and transposable elements, alcohol is well placed to affect intrinsic transcriptional programs of developing cells. Considering that the developmental consequences of early alcohol exposure so profoundly affect neural systems, it is not unfounded to reason that alcohol exploits transcriptional regulators to challenge canonical gene expression and in effect, intrinsic developmental pathways to achieve widespread damage in the developing nervous system. To fully evaluate the role of epigenetic regulation in alcohol-related developmental disease, it is important to first gather the targets of epigenetic players in neurodevelopmental models. Here, we attempt to review the cellular and genomic windows of opportunity for alcohol to act on intrinsic neurodevelopmental programs. We also discuss some established targets of fetal alcohol exposure and propose pathways for future study. Overall, this review hopes to illustrate the known epigenetic program and its alterations in normal neural stem cell development and further, aims to depict how alcohol, through neuroepigenetics, may lead to neurodevelopmental deficits observed in fetal alcohol spectrum disorders.

## PART ONE: NORMAL EPIGENETIC PROGRAM IN DIFFERENTIATING NEURAL STEM CELLS (NSCs)

### INTRODUCTION

When neural precursors begin their journey into specified, mature neurons they undergo much transcriptional re-programming. This involves the silencing of pluripotency genes that act to keep the cell in a primordial stage as well as the activation of neuron-specific genes that permit the morphological and functional capabilities of the mature cell. It comes as no surprise then that a host of chromatin remodeling proteins, including epigenetic machinery, undergo considerable transformation during this time. After all, to accommodate the transcriptional changes necessary for cellular specification, relevant DNA regions must undergo structural changes to either facilitate or hinder the accessibility of the loci to transcriptional machinery. In just the last decade, an unprecedented growth in our understanding of the molecular underpinnings of these structural changes has occurred. We have uncovered and expanded the investigation of several classes of epigenetic modifications from histone to DNA and more recently, non-coding elements of the genome which can also play a role in shaping which genes are expressed during the critical, developmental phases of neural maturation. Skepticism regarding the gravity of these epigenetic factors in normal mammalian development has been answered by the revelation that deleting critical enzymes, such as DNA methyltransferases, decreases the viability of offspring or results in embryonic lethality (Li et al., 1992; Okano et al., 1999). Likewise, mutations in the genes of other epigenetic machinery have been linked to developmental diseases such as the MeCP2 mutation in Rett syndrome, a disease which results in detrimental nervous system development (Amir et al., 1999; Guy et al., 2001). Together, this evidence suggests that epigenetic machinery not only plays a role but is required for the progression of normal neural development.

Much effort has been made to understand how epigenetic markers are altered during neuronal differentiation. Directed differentiation of neuronal fates from totipotent embryonic cells (*in vitro*) as well as live developmental study of mammalian animal models have shown that epigenetic transformation, in line with transcriptome reorganization, is robust and dynamic. Importantly, these changes occur in very cell (lineage)-specific ways and follow strict spatial and temporal cues. Altogether, we propose that an epigenetic program is necessary to drive the transcriptional profiles that differentiate a neural precursor. Here we present just a fraction of the hundreds of neural epigenetic targets that contribute to the development of a neuron. Particularly, we discuss these genes in the context of developmental signaling pathways known to be required for the specificity of mature neural cells-everything from cell cycle arrest to inhibition of neuronal apoptosis and the onset of neuron and glia differentiation. Throughout, we note that some genes are targets for multiple epigenetic modifications and that one modification often begets another. Indeed, the epigenetic drivers of neurodevelopmental pathways are complex and highly integrated with one another thus allowing external influences a host of downstream opportunities from a single starting position. From this vantage point it is easier to understand why developmental time points are so much more sensitive to external stimuli and how these early exposures can drive lasting change in a neural system (more of which will be discussed in part two).

### NEURAL STEM CELL DIFFERENTIATION: GLOBAL TRANSFORMATIONS

**FIGURE 1:**
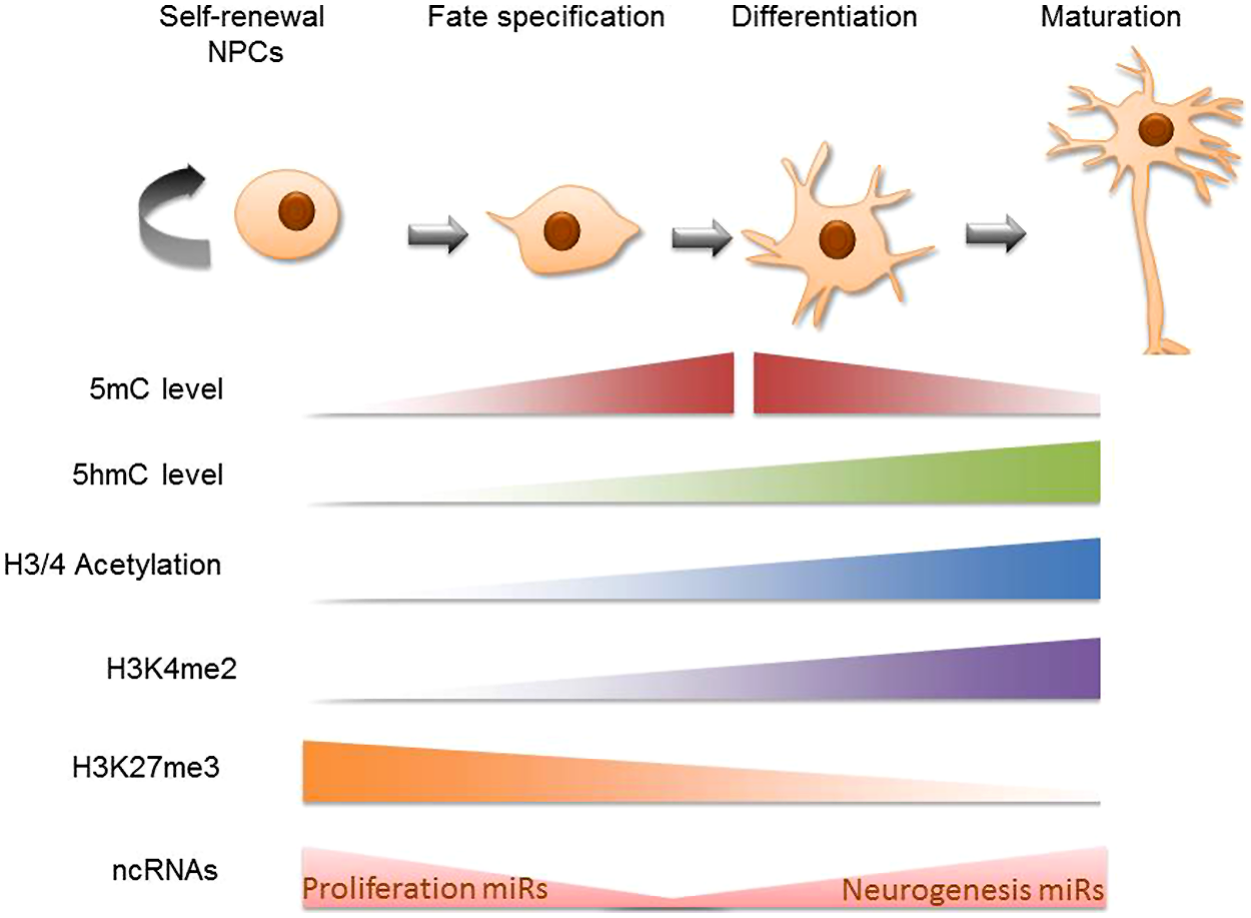
**Global epigenetic trends in neural stem cell differentiation.** Schematic diagram of cellular epigenetic program during neurogenesis. The top panel shows cell states during neurogenesis, from self-renewing neural progenitor cells (NPCs) to fate-determined neuroblasts, to differentiating and maturating neurons. The epigenetic programing is mapped in the bottom panel accordingly: cells gain 5 mC at the beginning of cell specification and sequentially gain 5 hmC at the beginning of cell differentiation; both 5 mC and 5 hmC accumulate during neuronal differentiation and maturation while at later stages of neuronal maturation, 5 mC levels decrease (Chen et al., 2013). Global trends in H3 and H4 acetylation have been traced *in vivo* to find that mature cells such as those in the mouse cortical plate are richer for these markers than the neural progenitor cells that preceed them (Cho et al., 2011). H3K4me2 is primarily acquired in the neural progenitor cell stage and becomes pronounced in the matured brain cell stage (Zhang et al., 2012). H3K27me3 has been shown to be negatively correlated with 5 hmC on intergenic regions during NPC differentiation (Hahn et al., 2013). Finally, MicroRNAs that support proliferative gene expression are diminished as self-renewable NPCs become specified neural precursors while pro-neurogenesis non coding RNA are upregulated during NSC conversion to a mature neuronal state (Meza-Sosa et al., 2014).

The earliest cellular commitment of a neural cell occurs when embryonic, totipotent stem cells become neural progenitors. During this time many investigators have noted global changes occurring in the epigenetic profile of these transformative cells. Histone acetylation increases among maturing neural progenitors *in vivo*, this is likely occurring to accommodate the increasing rates of RNA synthesis occurring in the cell (recall that acetylation of chromatin results in de-compression of DNA; Cho et al., 2011). Additionally, histone marks like H3K4me2 are predictably re-organized throughout the neural differentiation timeline on relevant genes. A high-throughput analysis revealed that H3K4me2 marks are acquired between the stages of pluripotent embryonic stem cells (ESCs) to neural progenitors and mature neurons on cell adhesion, synaptogenic, and neural transmitter signaling pathway genes (Zhang et al., 2012). The histone mark H3K27me3, on the other hand, has been found to decrease in the intergenic regions during neural progenitor cell (NPC) differentiation (Hahn et al., 2013). DNA methylation patterns have also been characterized in developing neural systems. Mainly, it has been shown that 5-methylcytosine (5 mC) is upregulated in neuroepithelial cells (NE) and rapidly downregulated during the specification of NE cells to mature neuronal populations (Chen et al., 2014). 5-Hydroxymethylcytosine (5 hmC; a derivative of 5 mC) patterns appear alternatively enriched at regions of active maturation compared to neuroprogenitor sites. Additionally, several high-throughput DNA methylation analyses of differentiating ESCs have reported that DNA methylation is altered on multiple genes on the path to neural progenitor conversion. These methylation shifts are bi-directional and include hypermethylation and hypomethylation (Singh et al., 2009; Cortese et al., 2011). Perhaps more important than cumulative levels of DNA methylation, however, are the recent findings that genomic landscapes undergo DNA methylation shifts such that regions previously methylated become hydroxymethylated while other un-methylated regions acquire methylation during this critical time of neural predisposition (Hirabayashi et al., 2013). The observation that neural gene clusters appear to acquire 5-hydroxymethylcytosine (5 hmC) during ESC to NPC differentiation led to the hypothesis that though 5 hmC does not directly up-regulate the genes that promote neural differentiation, the methylation intermediate may act as a “priming” mechanisms for the de-methylation which will eventually allow the expression of these genes (Tan et al., 2013). Many non-coding RNA transcripts are similarly altered during ESC conversion to neural progenitors (Iyengar et al., 2014), potentially impacting a host of complimentary mRNA. Briefly, microRNAs involved in provisioning self-renewal capacity to neural stem cells (NSCs; miR 134, 137, 25) are understandably reduced as NPCs undergo neural specification and lose their proliferative ability (Meza-Sosa et al., 2014). Conversely, miRs that support neurogenesis (miR 124, 9, let7) are upregulated during the developmental progression of NPCs to immature neurons. Finally, chromatin-remodeling proteins have been shown to undergo up to 30-fold changes during neural precursor specification (these can be entirely lineage specific; Juliandi et al., 2010; Weng et al., 2012). These protein complexes interact with and/or influence subsequent epigenetic modifications on the path to regulating neuronal transcriptomes. It is worth noting that studies of global epigenetic change during neural differentiation often come from two sources, cell populations analyzed in live developmental systems or cultured NSCs. The use of one versus the other can lead to contradictory conclusions regarding the nature of epigenetic change. As Cho et al. (2011) explains, this is not surprising given that epigenetic modifications are often products of external cues and *in vivo* extracellular environments have not yet been precisely recapitulated *in vitro*. Altogether, there is ample evidence to support that these epigenetic mechanisms (histone modification, DNA methylation, non-coding RNA elements, and chromatin remodeling proteins) contribute largely to the transcriptional re-programing that is required during stem cell commitment to neural lineages (**Figure 1**). Next we discuss particular gene targets and the integrative epigenetic modifications which guide them along the specification pathways that distinguish neural precursors.

### NEURAL STEM CELL DIFFERENTIATION: GENE NETWORKS AND EPIGENETIC REGULATION

#### (A) Cell-cycle regulation and pluripotency

Cell cycle regulators play an important role in developing NSCs. Primarily, they allow the self-renewal of neural progenitors and are ultimately responsible for the cell cycle arrest that occurs when a progenitor becomes post-mitotic (no longer able to self-renew). Both symmetric (self-renewing) and asymmetric cell division are important for the development of cortical cells in the CNS. The number of cell divisions of neuroprogenitors will determine the number of mature neurons in the brain. The propagation of symmetric cell division past the normal developmental schedule or, conversely, the premature arrest of the cell cycle can drastically alter the structure and, ultimately, lead to functional aberrations. To make sure that cell cycles adhere to preset schedules, epigenetic mechanisms are utilized to regulate the expression of pro-mitotic and pro-pluripotent genes. One example of epigenetic regulation at the cell cycle level can be observed during the G2 to mitosis phase. For this mitotic progression to occur, cdk2/cyclinA and cdk1/cyclinB must sequentially phosphorylate FoxM1, an important transcription factor for pro-mitotic genes (*Cycb, Cenpf*). The transcription factor SC1, which has been shown to recruit the type II arginine methyltransferase PRMT5, works to repress the pro-mitotic genes *Cyclinb* and *Bub1b*, thereby keeping cells in a proliferative state (Chittka et al., 2012). When SC1 is deleted, premature differentiation of neural precursors is observed. This is just one small part of the cell cycle at which epigenetic interference can work to influence the developmental status of a neuroprogenitor. In fact many more cell cycle regulatory genes have been ousted as epigenetic targets.

Another facet of neural development that aids in the “stemness” or the self-renewal property of a neural precursor involves a network of pluripotency-promoting transcription factors. Oct4 and Nanog share an overwhelming number of target genes, most of which promote the Inner Cell Mass (ICM) conditions from which ESCs are derived. They help maintain cells in a pluripotent state by either repressing or activating the expression of associated genes. They may also form a complex with Sox2 and together, regulate neighboring Sox elements involved in embryonic development. JARID1B, the H3K4me2 demethylase, has been shown to affect the expression of Oct4 and Nanog. Specifically, JARID1B plays a hand in suppressing the expression of these transcription factors as deletion allows Oct4 and Nanog expression to continue past their normal time course in an *in vivo* developmental model (Schmitz et al., 2011). The H3K9 demethylase JMJD1C has also shown a direct binding capacity to Oct4 (Wang et al., 2014a). Additionally, the Jarid family of proteins may not be acting alone as they have shown complex-forming capacity with polycomb repressor proteins (PcGs)-chromatin remodeling proteins that act to repress gene activation (Pasini et al., 2010). Histone demethylases therefore contribute to neural differentiation dynamically by inhibiting activating histone methylation marks and by recruiting proteins that catalyze repressive histone methyl marks. In cases where PcGs overlap on target genes with histone demethylases (reportedly 90%), it is unclear whether there exists competition between demethylases of repressive histone marks and PcGs conferring new repressive histone marks. Oct4, Nanog and Sox2 have also been identified in screens of differential methylation during neural differentiation, indicating that a DNA methylation reprogramming occurs in these genes at the onset of their quiescence (Kim et al., 2014). Nanog, for example, lost 5 hmC in the enhancer regions and gained 5 mC promoter methylation while displaying decreased expression (though it remains to be resolved whether gain of 5 hmC or loss of 5 mC is primarily responsible for the observed expression change). Finally, Sox2 has also been identified as a direct target of the long non-coding RNA RMST and the miRNA 200c (Peng et al., 2012). lncRMST misexpression can inhibit normal neural maturation by affecting the expression of pro-neural genes regulated by Sox2.

As stated earlier, these are only small fractions of the cellular and genomic cascades that govern the replication of a neural precursor. Many other genes and factors are at work beyond what is presented here. Also, it is likely that, as in the case of Sox2, genes governing cellular stemness are actually affected by a myriad of epigenetic factors, both direct and indirect. This suggests that the ultimate expression of the target cannot merely be attributed to one epigenetic mechanism, as is customarily investigated and described, but rather the sum of all their interactions. Such epigenetic–genetic mapping would be a welcome and useful undertaking toward a more complete understanding of the epigenetic governance of an entire cellular property.

#### (B) Neurogenesis/gliogenesis and cell survival

Of course the path to neuronal maturation does not end upon exit of the cell cycle. For a neuronal precursor to mature to a final state it must follow a pathway of neural specification. Since there are many different mature neuronal fates, each with unique morphological and functional specificities, it only makes sense that there would exist many distinct neural pathways driving each neuronal subtype. Here we will only focus on a few of the many cascades of genetic profiles that ultimately drive a mature neuron into existence. These have been selected to showcase the interplay of pro-neural genes and epigenetic mechanisms.

The initiation of transcriptional drivers of neuronal maturation often comes from an escape from a repressive action. These inhibitory signals must first be lifted in order for pro-neural genes to activate the maturation schemes of the neural progenitor. A major inhibitory signaling cascade that exemplifies this is the Notch1 pathway. The Notch1 pathway plays a big role in CNS development and, depending on the timing of its activation, can heavily influence the fate of multipotent CNS precursors (Yoon and Gaiano, 2005). Notch1 activation signals the upregulation of Hes family genes. Hes1 and Hes5 specifically, can act to repress the pro-neural genes *Mash1 (Ascl1)* and *Ngn1/2*. These pro-neural factors typically form complexes and act as transcriptional activators of downstream genes important for neuronal specification (more of which will be covered later). While Notch1 signaling is active, neuronal precursors are pushed toward self-renewal (itself necessary to maintain an appropriate progenitor pool size). Meanwhile, cells that manage to become post-mitotic upregulate Notch1 ligands which, through lateral inhibition, restrict neighboring cells from undergoing their own post-mitotic specification (Kageyama et al., 2008). This negative feedback loop, however, is in competition with the negative regulator(s) of Notch signaling- at least one of which has also been found downstream of post-mitotic gene cascades (Kaltezioti et al., 2010). In other words, neuronal differentiation both inhibits and partly promotes Notch activation/silencing in neighboring cells.

Another factor in the neurogenesis/stemness equation is the presence of the transcription factor Pax6 – a regulator of NSC maintenance and neurogenesis genes. Pax6 interjects with Notch signaling by pairing with Ngn2 to promote neurogenesis. Together, they override the inhibitory action of Notch-mediated Hes1 (Sansom et al., 2009). Pax6 also plays a Notch-independent role in neurogenesis by inhibiting the expression of the pluripotency genes *Oct4* and *Nanog* (Zhang et al., 2010). Finally, several important neurodevelopmental transcription factors such as Sox2 and Pax6 exhibit ZEB-dependent expression (Du et al., 2013). ZEB family proteins act as transcriptional repressors for competing signaling cascades which seek to drive stem cells away from ectodermal (neural) lineages, namely BMP signaling (Postigo, 2003).

Some of the same pathways that direct neurogenesis play a hand in gliogenesis as well. Notch1 for example (likely through Hes1/5 and the downstream effector Dll1) interacts with the FGF-mediated Sox9 to promote astroglial fates (Grandbarbe et al., 2003; Wu et al., 2003; Bani-Yaghoub et al., 2006; Esain et al., 2010). The FGF signaling pathway, alternatively, can contribute to the formation of oligodendrocyte progenitors (OLP). This specification is probably due to the expression of the *Olig2* gene, which is expressed under the combined signaling of FGF and SHH (Esain et al., 2010). Additionally, FGF-regulated Sox9, when paired with Sox10, has been shown to aid in OLP survival and migration (Finzsch et al., 2008). Having presented just a faction of the many developmental signaling pathways that interact to regulate neuronal and glial fates, it becomes apparent that these signals are carefully poised to converge in space and time to drive a specific lineage. As such, deviations from these thresholds, no matter how small, can alter a signaling network enough to change the trajectory of a neural precursor. In other words, even small disturbances in these delicate signaling networks can produce a sort of “domino” effect by which lasting neurodevelopmental changes are propagated in an organism. In this vein, let us next consider the opportunities or “windows” that exist in these networks for epigenetic regulation and indirectly, for external input to propel developmental change.

Beginning with the Notch pathway, NSC differentiation analysis has identified both *Notch1 and Hes5* (along with a handful of other downstream Notch1 genes) as targets of differential DNA methylation (Kim et al., 2014). Specifically, the expression of these Notch-related genes displays some dependency on the methylation status of their promoter and/or gene body. Additionally, the histone modifiers SIRT1 and JMJD2B have been shown to affect the expression of Hes1 and Notch1, respectively, in models of neural progenitor differentiation (Hisahara et al., 2008; Das et al., 2013). At least in the case of Notch1, the histone demethylase JMJD2B acts on Notch1 expression by regulating the presence of the repressive histone mark H3K9me3 on the gene promoter. The pro-neural genes *Mash1* and *Ngn1* are not only repressed through active Notch signaling but also serve as direct targets of the Sox2-regulated miRNA let-7i (Cimadamore et al., 2013). This miRNA sequestration results in decreased neuroprogenitor proliferation and neurogenesis similar to that of Sox2-defficient precursors (recall that Mash1 and Ngn1 are downstream targets of Sox2). Ngn1 also serves as a target of the polycomb repressive complex (PRC) 1 and 2 (Hirabayashi et al., 2013). As discussed above, the transcription factor Sox2 is susceptible to both lncRNA and miRNA in addition to exhibiting differential methylation patterns during neural commitment. Additionally, the transcription factor has a promoter binding capacity for the histone 2B ubiquitinylase USP22, which in turn alters the recruitment of histone 3 methylation marks and ultimately leads to Sox2 repression, a function necessary for stem cell differentiation (Sussman et al., 2013). USP22 can also form a complex with the histone deacetylase SIRT1, which serves a similar repressive action on Sox2. There is evidence that some of the transcriptional regulators (inhibitors) of pluripotency factors also exist under the regulation of epigenetic machinery. The master transcription factor Pax6 which suppresses the stemness factors Oct4 and Nanog during ESC conversion to neural progenitor, is a target of the miRNA 96 family (Du et al., 2013). The repression of Pax6 by miR-26 members was experimentally confirmed and inhibited the differentiation of stem cells into neural precursory lineages exclusively. Finally, the ZEB transcription factor family, which is critical for repression of the competitive epidermal BMP signaling, has been isolated as a target of the miRNA 200 family (Du et al., 2013). This miRNA-mediated repression of ZEB is likely poised at the commitment of ectodermal precursors to either neural or epidermal fates. BMP (epidermal) repression via this epigenetic mechanism indeed swayed ESC populations toward neuroectodermal fates. Interestingly, early attempts to reshape the epigenetic landscape of important fate-determining pathways like Notch1 with epigenetic modifiers have proved unsuccessful (Reichrath and Reichrath, 2012). It is likely that as further investigation with more targeted approaches and diverse cell populations will yield promising results that will strengthen our understanding of these signaling pathways and their vulnerability to epigenetic influence.

We have now covered some of the major pathways that allow neural precursors to both self-renew and differentiate into more committed cells. We have discussed that this process involves the precise activation of pro-neural transcriptional networks as well as the timely de-activation or suppression of competing influences. Some of these competing pathways are aimed at repressing the maturity of a cell while other pathways work to drive maturing cells toward non-neuronal trajectories. Interestingly, we have seen that many genes play roles in multiple pathways and that neurogenesis/gliogenesis and their specification are likely the overall effect of converging networks and multiple contributing factors. Also, epigenetic mechanisms are likely involved in the intrinsic schedule that directs normal neural development. For example, differentiation cues in a stem cell can trigger DNA methylation re-distribution/conversion, histone modification or non-coding RNA binding. Like the integrative nature of the neural differentiation transcriptome, epigenetic factors are likewise heavily intertwined. In other words, one gene can be affected by multiple epigenetic mechanisms and it is unsurprising that sometimes the onset of one modification can recruit other alterations both on the same locus and/or in nearby regions. Early neural commitment is not the terminal point of the neural differentiation program. Before we address the pathways that further specify and finalize mature neuronal attributes we will first address a small portion of the pathways that are utilized for the maintenance and survival of committed neural precursors.

During development, many neurons undergo programmed cell death. Neurons that are “engaged” with one another, however, are typically spared. Of particular importance in neuronal survival is the PI3K-Akt cascade of the BDNF pathway, which can induce the transcription of either pro-survival or pro-apoptotic genes in a BDNF-dependent manner (Brunet et al., 2001). BDNF is a neurotrophin which, through TrkB activation, can trigger a variety of downstream cascades ultimately resulting in the transcription of survival factors like NFK-B and CREB (Romashkova and Makarov, 1999). Conversely, in the absence of BDNF, genes like the pro-apoptotic members of the Bcl-2 family can be upregulated and promote apoptosis (Brunet et al., 2001). Typically, the channel-gated accumulation of intracellular calcium upon neuronal communication triggers the initial activation of the BDNF cascade (either through PLC-g, CaM kinases, or the PI3K-Akt pathway) each independently capable of driving the nuclear transcription of BDNF and other survival genes (Marini et al., 2004). BDNF produced from these initial reactions can thus come back as a ligand for further TrkB activation. TrkB activation by neurotrophins can recruit PI3K to the inner surface of the plasma membrane where they produce phospholipids that recruit the kinase Akt. PDK1-mediated phosphorylation of Akt serves as an activating event which further allows Akt to act on a variety of downstream targets. For example, the unphosphorylated BAD protein is bound to the pro-survival factor Bcl-xL inhibiting it from promoting cell-survival. Upon neurotrophic Akt activation, BAD is phosphorylated and unbound from Bcl-xL, freeing it to promote survival (Datta et al., 2000). Factors that regulate apoptotic machinery and promote cellular survival are important to ensure proper neural development. Deviations from the intrinsic neural schedule of the expression of these genes can thus be detrimental to the overall architecture of the brain.

Epigenetic regulation of the BDNF cascade that promotes neuronal survival can be achieved through the BDNF gene itself. A natural antisense transcript for BDNF has been reported to repress BDNF expression *in vivo* (Modarresi et al., 2012). BDNF expression has also been increasingly tied to promoter DNA methylation in various models of neurological disease, indicating that even under normal developmental conditions, BDNF promoter methylation may be significantly responsible for neurotrophic levels (Ikegame et al., 2013). Activity-dependent changes in promoter methylation of the BDNF gene (5 mC, CpG methylation) are also thought to mediate the release of a repressive chromatin remodeling protein (mSin3A) from the promoter thereby providing anther epigenetic mechanism of BDNF regulation (Martinowich et al., 2003). The Akt1 gene exhibits similar methylation-dependent transcriptional regulation. During ESC differentiation, the gene is upregulated and this increase is reportedly correlated with the acquisition of intragenic 5 hmC (Kim et al., 2014). Downstream of Akt, CREB has revealed sites in its activating region where the CREB-binding protein (CBP) can acetylate lysine residues and in so doing, modulate CREB-mediated gene expression (Lu et al., 2003). Finally, the anti-apoptotic gene Bcl2 which promotes neural cell survival has been identified as a target of the miRNA 497 in some studies of neural insult, indicating that externally regulated neuronal cell death is at least partially achieved through epigenetic regulation of pro-survival transcripts (Yadav et al., 2011). As in the case of pluripotency and neurogenesis, multiple levels of epigenetic regulation may converge on a single gene. The ultimate regulatory action is thereby dependent on the sum of all these influences, which may act in similar or contradicting directions. Additionally, the onset of one epigenetic modification can often trigger sequential acquisition of further changes. A thorough understanding of the factors that dictate epigenetic change in the developing nervous system are still far beyond reach though it is clear that external impacts make use of epigenetic machinery to induce transcriptional and phenotypic change in the brain. Next we address the latent stages of neural development. After neuronal/glial commitment cells of the nervous system undergo transcriptional changes to further direct the specialized cell they will become for the remainder of their lifespan. These changes include migration, neurite outgrowth, and a host of synaptic preparations and refinements-some of which are never fully static and continue to evolve throughout adulthood.

#### (C) Late-stage neuronal specification and synaptic plasticity

After neurogenesis, maturing precursors continue to experience the fluctuation of a progressive trancriptome. This serves to accommodate the changing needs of a cell to acquire specific traits like proteins that would become receptors of electrical and chemical signals from neighboring cells. For example, cortical neuron specification can occur from radial glia precursors. The expression of the transcription factor Pax6 allows radial glia to produce both neuronal and glial precursors. The downstream upregulation of the transcription factor Tbr2 begins to negatively regulate the expression of Pax6 restricting radial glia production to only neuronal fates. Further, the onset of Tbr1 expression negatively regulates Tbr2, conferring corticogenesis (Englund et al., 2005). Further specification of mature cortical subtypes is controlled by distinct combinations of downstream genes. Subcortical projection neurons for example, have been linked to the expression of Fezf2 and Ctip2 sequentially (Leone et al., 2008). Alternatively, Satb2 likely regulates the fate of cortico-callosal projection neurons by repressing the aforementioned subcortical Ctip2 cell identity pathway (Alcamo et al., 2008). Many more specialized classes of cortical neurons exist and are regulated by a variety of genes. The cues directing the activation and inactivation of these key cell specification factors likely come from the microenvironment surrounding the precursor at a specific time point.

Another example of a highly specialized neuronal maturity cascade has been studied in dopaminergic neurons. Neural progenitors isolated from the ventral midbrain show a dependence on Ngn2 and Nurr1 (Nr4a2) for the production of morphologically mature and functional dopaminergic neurons *in vitro* (Andersson et al., 2007). Further, the transcription factor Pitx3 has been implicated in DA neuron survival and production of AHD2, an enzyme which produces retinoic acid and is present only in a subset of DA neurons (Chung et al., 2005). Retinoic acid binds and activates retinoic acid receptors which in turn may regulate the expression of tyrosine hydroxylase and influence the production of dopamine (Jeong et al., 2006). Corticogenesis and dopaminergic specification are both examples of the later-stages of neural development where neural progenitors have already been committed to neuronal fates but require lots of “fine-tuning.” These types of specialized neurons can take a long time to fully mature as many genes (not discussed here) will continue to regulate phenomena such as the production and migration of synaptic receptors and the appropriation of morphological properties conducive to the functions of the cell.

Epigenetic mechanisms have demonstrated the ability to regulate the timely expression of these fate-determining genes. As previously mentioned, the neuron-conferring transcription factor Pax6 is targeted by microRNA and has also been shown to express differential DNA methylation in accordance with neuronal maturity (Kim et al., 2014). Further down the cortical specification cascade, Ctip2 has been shown to bind histone modification enzymes to aid in transcriptional repression (Marban et al., 2007; Tan et al., 2012). Finally, the *Satb2* gene, thought to play a role in specification of cortical-callosal projections, is downregulated in the absence of the histone methyltransferase ESET, implying that this histone enzyme is required for normal neural development, likely through the regulation of one or more corticogenesis pathways (Tan et al., 2012). The role of dopaminergic neuron late-stage specification has also been epigenetically investigated. Histone modification, for example, has been shown to regulate the expression of Nurr1 and its downstream targets Pitx3 and Dlk1 – all essential in the synthesis, metabolism, and transport of dopamine (van Heesbeen et al., 2013). Similarly, epigenetic elements have been discovered in the regulation of specification genes of other mature neuronal types (Boshnjaku et al., 2011; Banerjee et al., 2013).

Some properties of cellular structure and function are continually fluid, or plastic, in the brain. In fact, it is cellular plasticity which allows neurons the ability to aid in human learning, cognition, and memory (among others). Thus, the constant disposition of certain developmental factors allow for the adaptation of a cell to changing demands throughout life. An example of paramount importance is synaptic plasticity or the ability of the neuron to change the components of its neurotransmitters and/or receptors to adapt to the strength of incoming/outgoing signals. The activation of NMDA receptors in the hippocampus during long-term depression (LTD) and long-term potentiation (LTP), both of which contribute to learning and memory in the adult brain is just one of the many ways that lasting plasticity is achieved. NMDA Receptors are ionotropic receptors of the excitatory neurotransmitter glutamate. Activation of these receptors triggers the influx of extracellular Ca^2+^ such that they are able to bind to the calcium-dependent protein calmodulin at the cytoplasm. From here calmodulin is able to translocate calcium so as to reach intranuclear calcium-calmodulin dependent kinases. Specifically, CaMKIV have been shown to be critical for the phosphorylation of CBP and downstream CREB/CBP-dependent transcription (Impey et al., 2002). Interestingly, it has been reported that in the hippocampus, CREB phosphorylation (activation) is attainable via endogenous intranuclear calcium and CaM kinase stores, independent of calmodulin (Hardingham et al., 2001). Synaptic action potentials acting through NMDA receptor-induced calcium transients thus regulate genes capable of re-shaping the synaptic landscape. Furthermore, duration of calcium transients are thought to dictate the transcriptional response of CBP/CREB (Chawla and Bading, 2001) and in this manner determine whether LTP or LTD of synapses is achieved (Luscher and Malenka, 2012). Nr4a2 (Nurr1) is an important transcriptional regulator of dopaminergic lineages and a target of CREB/CBP (Vecsey et al., 2007). The neuronal survival neurotrophin BNDF has also been shown to be under the transcriptional regulation of CBP/CREB (West et al., 2001; Hardingham et al., 2002). Many other genes relevant to neuronal growth and maintenance have been implicated in CBP/CREB transcriptional control including immediate early genes (Cole et al., 1989).

Much remains unknown about the role of epigenetics in the late-stage refinement of a maturing neuron. Puckett and Lubin summarize various classes of known epigenetic modifications which may occur in fully mature, adult neurons such as those in the hippocampus involved in long term potentiation and other experience-driven molecular responses. To point out just a few, the transcriptional repressor CREB2, an important player in memory-related synaptic plasticity, is targeted by a type of non-coding RNA (piwi-associated RNA; Rajasethupathy et al., 2012). The CBP itself has been characterized as a recruiter of histone acetyltransferases to gene promoters thereby stimulating transcription (Bannister and Kouzarides, 1996). Downstream targets of CBP/CREB also exhibit epigenetic modifications which together with CBP/CREB can account for expression levels during activity-dependent synaptic plasticity (Guan et al., 2009). Beyond individual gene modifications, large-scale DNA methylation and histone acetylation have also been shown to be critical for memory consolidation and synaptic activity in the hippocampus (Vecsey et al., 2007; Miller et al., 2008). HDACs in particular have been attributed to reduced dendritic spine number and synapse number (Guan et al., 2009). This disposition of fully mature neurons to epigenetic change is particularly important when considering environmentally acquired epigenetic modifications. This means that even fully mature cells can be susceptible to aberrant external stimuli and thus supports the role of epigenetic processes in mediating not only developmental but late-onset disease. Beyond intracellular calcium fluxes and CBP/CREB elements, not much is known about the mechanisms translating neuronal activity into epigenetic regulation. Progress continues to be made toward the understanding of epigenetic regulation of synaptic plasticity networks, such as the involvement of histone acetyltransferases on NFk-B targets during memory consolidation (Stilling et al., 2014) and the interplay of histone 3 acetylation and phosphorylated CREB on the promoter of the gene encoding PSD-95 during reward learning (Wang et al., 2014b). Still, it remains important to investigate the molecular basis of epigenetic response to altered neural stimuli (the other half of the equation), which surely spans beyond the current confines of calcium response elements. This will be essential to forward our understanding of the genes provisioning synaptic plasticity and our ability to intervene in activity-dependent disease.

### CROSSTALK

**FIGURE 2:**
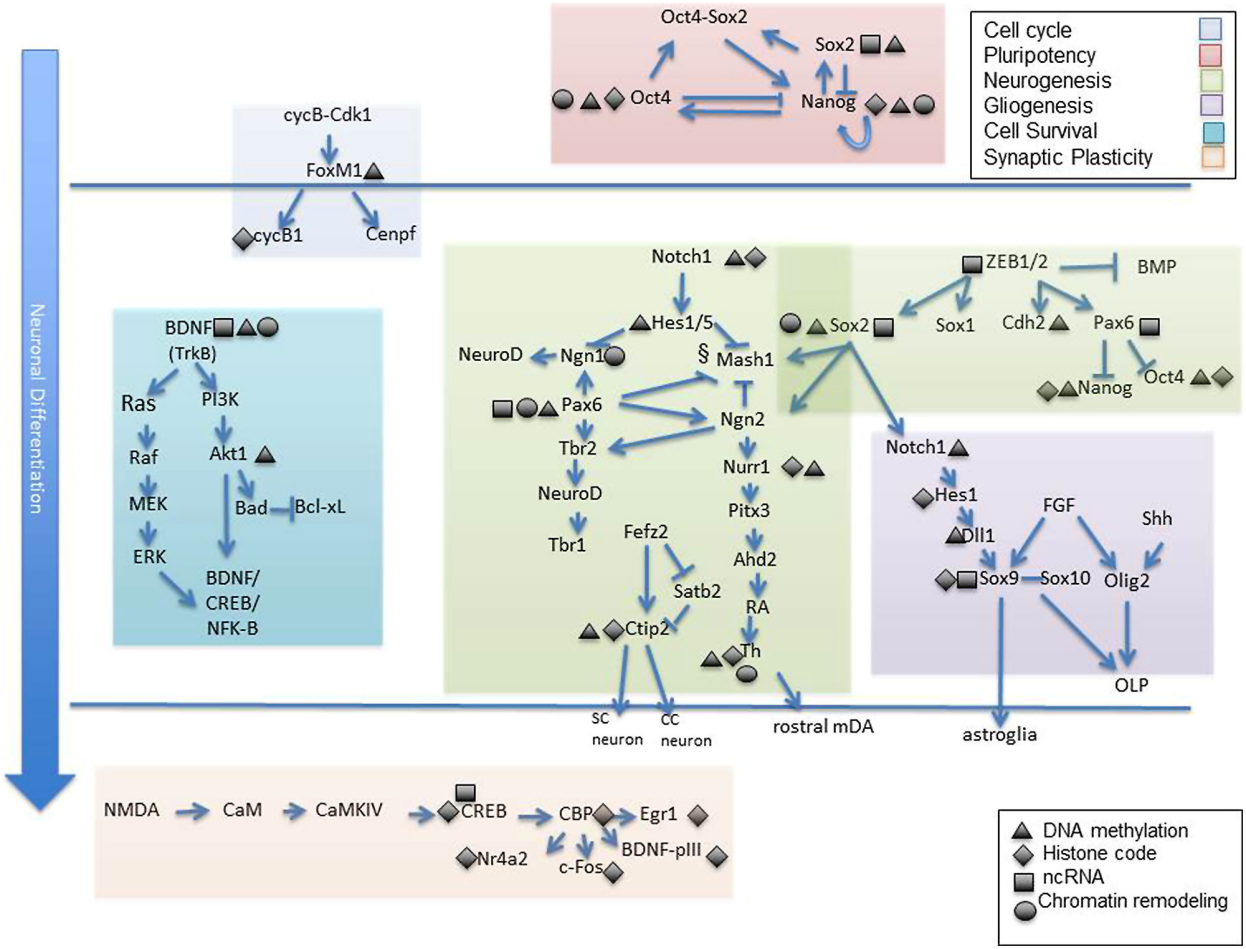
**Epigenetic targets of neuronal differentiation.** Select gene cascades are presented which correspond to various biological processes known to mediate neuronal commitment and specification from early to late stages. Among these are genes regulating cell cycle progression (shaded blue), pluripotency (shaded pink), neurogenesis (shaded green), gliogenesis (shaded dark purple), cell survival (shaded turquoise) and synaptic plasticity (shaded orange). Gene targets know to exhibit epigenetic sensitivity are depicted as such via symbolic representation (black triangle, DNA methylation; black diamond, histone modification; black rectangle, non coding RNA, black cirlce; chromatin remodeling complex). Individual reference to each gene is made in the text. OLP, oligodendrocyte progenitors; SC, subcortical neuron; CC, corticocortical neuron.

While here we have primarily discussed epigenetic modifications that contribute to neural cell development as singular contributions, a more realistic scenario is that epigenetic modifications influence each other and that the ultimate transcriptional outcome is the sum of all these interactions. One example, established by Meissner et al. (2008) is the finding that histone methylation marks are strongly correlated with DNA methylation in a model of differentiating stem cells. Specifically, the activating acquisition of intragenic 5 hmC during neural differentiation is closely tied to loss of H3K27me3. Moreover, this epigenetic shift was concomitant with loss of promoter Polycomb marks which, when induced, drove cellular differentiation away from the neural fate (Hahn et al., 2013). These are just some of the many instances of epigenetic dependence and/or interaction. Jobe et al. (2012) outlines other experimental examples of such epigenetic interrelations which occur in NSC fate specification. Finally, **Figure 2** attempts to visually represent some of the studies reviewed herein and to offer a conceptual understanding of the many gene targets affected by epigenetic alteration during neural specification, development, and synaptic plasticity. The vulnerability of gene targets in the neurodevelopmental cascade to epigenetic change leave one very important implication. Though epigenetic mechanisms regulating neurodevelopmental genes may have an intrinsic component, it is also highly likely that epigenetic modification is a response to an environmental input (Jaenisch and Bird, 2003; Feil and Fraga, 2011). As such, genes critical to structural and functional neuronal specification serve as “targets” for external factors which may compromise the normal epigenetic developmental program. Some of these external signals have been identified as air contaminants, fetal nutritional components, and substances of abuse (Heijmans et al., 2008; Guerrero-Preston et al., 2010; Esposito et al., 2014).

## PART TWO: ALCOHOL (ENVIRONMENTAL) INTERFERENCE OF THE NEURODEVELOPMENTAL EPIGENETIC PROGRAM

### ALCOHOL DYSREGULATION OF NEURAL DEVELOPMENTAL PROGRAMS

**FIGURE 3:**
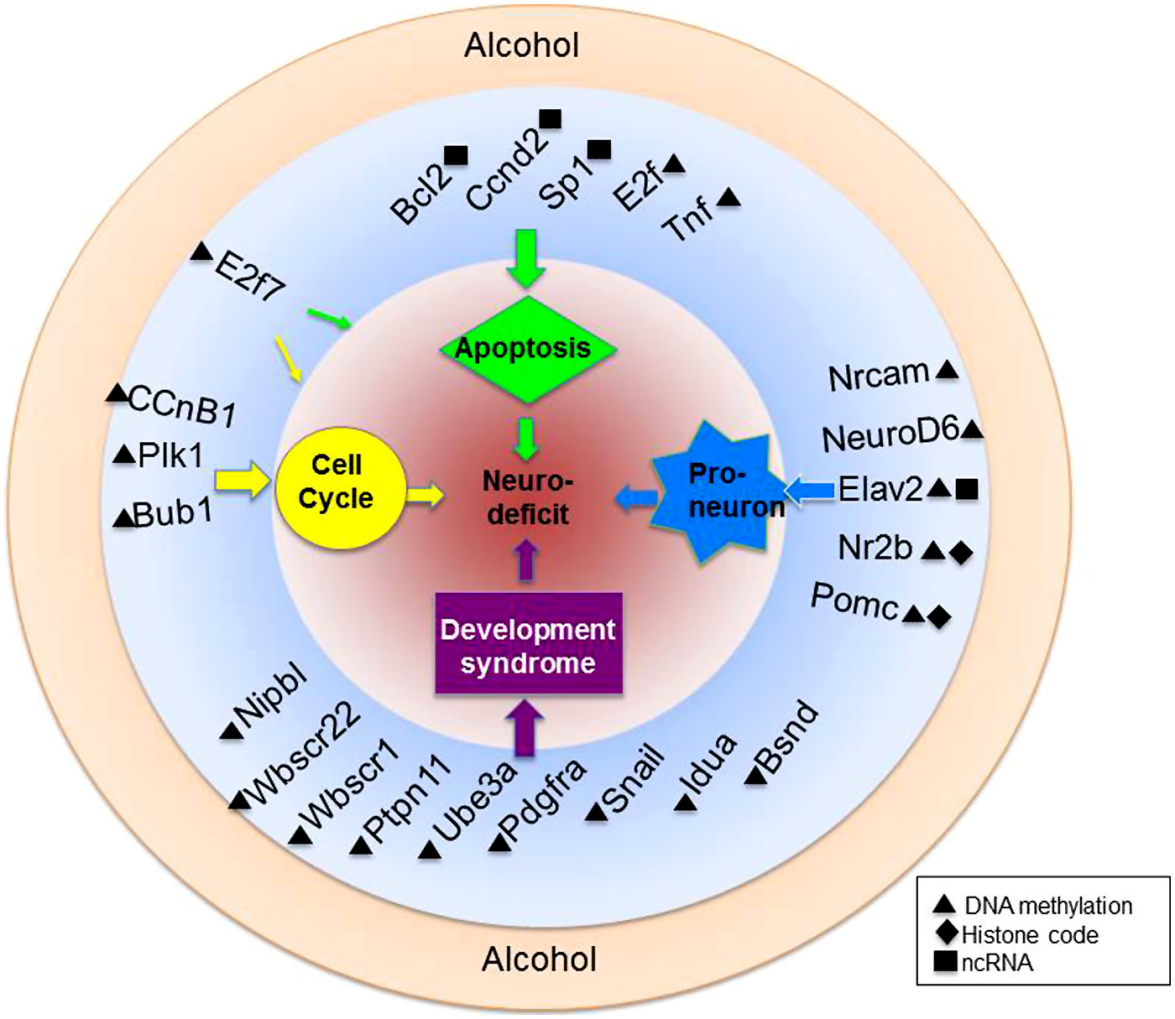
**Alcohol-mediated epigenetic targets of neural development.** Alcohol is a teratogen with known capabilities to alter the epigenome. Highlighted here are just a fraction of genes within various biological pathways of known vulnerability to ethanol-mediated epigenetic alteration. Also depicted are genes associated with known developmental syndromes and their epigenetic alteration. Genes are more specifically discussed and referenced in the text.

Because the transcriptional programs of a maturing cell are under epigenetic control, there is a pathway for environmental regulation of cellular maturation as well. Alcohol, for example has been shown to inhibit the differentiation of NSCs in culture. Compromised cellular growth, migration, and cell viability have been reported in models such as these (Zhou et al., 2011; Campbell et al., 2014). Additionally, a host of genes have been shown to be deregulated in precursory neurons by alcohol exposure (Sanchez-Alvarez et al., 2013) **Figure 3** summarizes three important physiological processes known to be targeted by alcohol in a gene-specific manner.

First, the importance of a tightly controlled cell cycle transcriptome was described earlier. NSCs treated with ethanol exhibit cell cycle delays, reduced NSC proliferation and increased DNA fragmentation (Anthony et al., 2008; Hicks et al., 2010). Some of the genes involved in cell cycle progression are unsurprisingly transcriptionally altered by ethanol. Of these, a few concomitantly exhibit epigenetic alteration. For example, DNA hypermethylation was detected on *CcnB1, Cdc20, Bub1,* and *Plk1* in the presence of alcohol (Hicks et al., 2010). In another NSC model, the presence of ethanol blocked the intrinsic hypermethylation of the cell cycle genes *Adra1a, Tnf, Pik3r1,* and *Sh3bp2* that is observed during differentiation (Zhou et al., 2011). The cell cycle genes for cyclinD1 and cdk6 have also been identified as targets of the alcohol-induced miR 34a in lung cancer cells (Sun et al., 2008). It would be interesting to investigate whether miR 34a or other miRNA families similarly target cell cycle regulatory genes in neuronal models.

Another cellular pathway affected by early ethanol exposure is cell survival. Neural progenitors exposed to alcohol *in utero* exhibit marked increases in cellular loss and markers of cell death (Ikonomidou et al., 2000). There appear to be several ways that ethanol can interfere with normal neuronal survival cues. In cultured granule cells, ethanol suppresses the endogenous miR29b, thought to protect against apoptosis, or cell death via the SP1 cascade of PKR phosphorylation (Qi et al., 2014). Other pathways that have been investigated indicate that ethanol acts through Bcl-xL (Bax) as deletion of the gene inhibits the ethanol-mediated apoptotic response (Young et al., 2003). Like SP1, several genes involved in neuronal survival have demonstrated some degree of epigenetic regulation. The miRs 497 and 302b are both elevated in the presence of ethanol and target the cell survival genes *Bcl2* and *Ccnd2* (Yadav et al., 2011). Interestingly, ethanol appears to exert a bidirectional effect on miRs-upregulating some miRs while suppressing others. Ultimately, it is proposed that alcohol initiates a physiological response like apoptosis in neural progenitors only if the miRs targeting antiapoptotic factors out-compete the miRs targeting apoptotic factors (Sathyan et al., 2007). Finally, we have previously reported that DNA methylation is altered by alcohol on the survival genes *E2f7* and *Tnf* (Hicks et al., 2010; Zhou et al., 2011). Reactive oxygen species (ROS) play a role in neuronal apoptotic pathways and are reportedly upregulated by ethanol exposure in a human neuronal cell line. Interestingly, treatment with the HDAC inhibitor Trichostatin A was neuroprotective and aided in the reduction of ROS (Agudelo et al., 2011). These results indicate that ethanol-mediated oxidative stress acts at least partially through histone modification enzymes.

In addition to the timely progression of cell cycle program and tightly regulated neuronal survival transcriptome, neuronal precursor maturation relies heavily on the appropriate differentiation signals we refer to as proneural cues. Several published reports have identified that exposure of ethanol to neural precursors delay or divert the intrinsic developmental trajectory (Chen et al., 2013; Sanchez-Alvarez et al., 2013; Veazey et al., 2013). Ethanol has even been shown to inhibit specific morphological aspects crucial to neuronal differentiation such as axon outgrowth and migration (Zhou et al., 2001; Chen and Charness, 2008). Part 1 of this chapter outlined some of the differentiation pathways which contain genes known to be targeted by epigenetic regulation, from early differentiation cues, to later-stage specification markers. Once again the question remains whether any of these genes are epigenetically altered in an alcohol-dependent manner. These are the genes that we will want to probe for their role in alcohol-mediated developmental disease such as fetal alcohol syndrome-those known to dysregulate normal, neural developmental programs. The *Nr2b* gene, which encodes a protein subunit of the NMDA receptor, has exhibited alcohol-induced histone 3 lysine 9 acetylation in conjunction with increased expression (Qiang et al., 2011). Nr2b upregulation has been linked with alcohol dependence-related hyperexcitability though the epigenetic action of alcohol on this locus has not been thoroughly examined in development. Several other neurotransmitter receptor genes have been identified as epigenetic targets of alcohol exposure in NSCs including the AMPA3 gene *Gria3*, which undergoes promoter methylation alterations (Zhou et al., 2011) and the brain cannabinoid receptor 1 gene which is downregulated following the induction of the miR26b (Stringer et al., 2013). The prodynorphin promoter reportedly undergoes alcohol-mediated downregulation related to histone methylation and acetylation (D’Addario et al., 2011). Additionally, prodynorphin SNPs have exhibited differential methylation patterns in the post-mortem brains of alcohol-dependent patients (Taqi et al., 2011). Finally, the proopiomelanocortin (POMC) gene is genetically and functionally altered by fetal alcohol exposure and these changes are lasting into adulthood in beta-endorphin producing POMC neurons (Bekdash et al., 2013). Genetic alterations were accompanied by hypermethylation of the gene and more importantly, were normalized when fetal alcohol exposure was paired with gestational choline supplementation. Not only does a greater understanding of the epigenetic mechanisms of developmental gene regulation allow us to fully grasp intrinsic neurodevelopmental processes, it provides opportunities for therapeutic intervention of neurodevelopmental diseases with known or suspected epigenetic etiologies.

We have only provided a brief look at some of the canonical pathways and genes affecting neurodevelopment that are known targets of alcohol. Much remains to be uncovered about the role of alcohol in epigenetic dysregulation of other pathways critical to neuronal maturation. The pluripotency genes *Oct4 (Pou5f1), Sox2,* and *Nanog* for example, have demonstrated an ethanol-specific delay of downregulation in NSC models (Ogony et al., 2013; Sanchez-Alvarez et al., 2013). Though we now know that each of these genes displays some degree of epigenetic sensitivity, it remains to be seen whether alcohol specifically acts on these transcripts through an epigenetic mechanism. It is likely that many other genes across a plethora of developmental cascades will exhibit association with epigenetic modification in the coming years. Some likely candidates which are dually but independently altered by ethanol and epigenetic modifiers can be found in **Table 1** and cross a variety of biological roles. These genes may serve as possible origins of the neurodevelopmental deficits observed in fetal alcohol models which include craniofacial dysmorphology, growth deficits, and intellectual disability. By altering the epigenetic code of primitive neural cells, environmental affectors such as ethanol are capable of re-shaping the course of normal, neural development to drive lasting, structural and functional changes. While the mechanisms by which environmentally driven epigenetic modifications act on transcriptional machinery are still being worked out, it is important to strive for a deeper understanding of the genetic/epigenetic dynamic.

**Table 1:** Neurodevelopmental genes as dual targets of alcohol and epigenetic modifiers.

Gene	Class	Alcohol effect on gene expression	Epigenetic modifiers effect on gene expression
Oct3/4	Pluripotency	↑ (Arzumnayan et al., 2009; Ogony et al., 2013; Sanchez-Alvarez et al., 2013)	↑ by 5-azacytidine, TSA+5-aza-2′-deoxycytidine, LSD1 small inhibitors, VA/5-aza-2′-deoxycytidine + dTALEs (Tsuji-Takayama et al., 2004; Ruau et al., 2008; Wang et al., 2011; Bultmann et al., 2012)
Sox2	Pluripotency	↑ (Arzumnayan et al., 2009; Sanchez-Alvarez et al., 2013)	↑ by 5-azacytidine, LSD1 small inhibitors (Tsuji-Takayama et al., 2004; Wang et al., 2011)
Nanog	Pluripotency	↑ (Arzumnayan et al., 2009)	↑ by 5-azacytidine, TSA + 5-aza-2′-deoxycytidine (Tsuji-Takayama et al., 2004; Ruau et al., 2008)
SSEA-1	Pluripotency	↑ (Arzumnayan et al., 2009)	↑ by 5-azacytidine (Tsuji-Takayama et al., 2004)
Dlx2	Pro-neural	↑ (Veazey et al., 2013)	↓ by TSA (Ignatius et al., 2013)
Nestin	Pro-neural	↑ (Veazey et al., 2013)	↑ by 5-aza-2′-deoxycytidine/TSA/RA (Han et al., 2009)
CCnB1	Cell-cycle regulation	↓ (Hicks et al., 2010)	↓ by TSA + SAHA w/silibinin (Mateen et al., 2012)
CCnD1	Cell-cycle regulation	↓ (Hicks et al., 2010)	↓ by honokoil (Singh et al., 2013)
Pttg1	Cell-cycle regulation	↓ (Hicks et al., 2010)	↑ by p300 (Li et al., 2009)
hdac4	Neural maturation	↑ (Vangipuram and Lyman, 2012)	↑ by TSA (Chu et al., 2008)
Ache	Neural maturation	↓ (Vangipuram and Lyman, 2012)	↑ by 5-aza-2′-deoxycytidine (Bartolucci et al., 1989)

*Genes involved in several biological pathways including neural differentiation exhibit dual sensitivity to alcohol and epigenetic modifiers. Some of these sensitivities have been tested in neural stem cells while others have been demonstrated in cancer cells. The disposition of these genes to expression changes by epigenetic modification and alcohol along with their biological relevance in cell development make them prime candidates for more thorough investigation in neurodevelopment and neurodevelopmental disease etiology. TSA, trichostatin A; VA, valproic acid; LSD1, lysine-specific demethylase 1; dTALE, designer transcription activator-like effector; RA, retinoic acid; SAHA, suberoylanilide hydroxamic acid.*

Lastly, there are genes which have been specifically identified in neurodevelopmental disease etiology (such as autism and fetal alcohol syndrome). These genes exhibit epigenetic sensitivity, though the causal nature of the epigenetic mechanisms remains to be scrutinized. Some of these genes have been previously outlined (Resendiz et al., 2013). Briefly, FASD models have identified Pten, Nmnat1, Slitrk2, and Otx2 as targets of ethanol-directed miRs. Additionally, the imprinted genes *Ube3a* and *Dlk1* have exhibited lasting differential methylation (Laufer et al., 2013). Other diseases with phenotypes overlapping with FAS, such as intellectual deficits rooted in neurodevelopmental aberration, have been investigated. An increase in 5 mC and reduction of 5 hmC at the A2AR receptor gene was identified and associated with transcript reduction in the putamen of Huntington’s disease patients (Villar-Menendez et al., 2013). Rett syndrome and autism patient cohorts have both revealed mutations in the demethylase enzyme JARID1C, thought to regulate transcriptional repression (Adegbola et al., 2008; Wynder et al., 2010). The epigenetic importance of developmental genes mediating or involved in disease etiology has become particularly apparent as reports of the longevity and heritability of epigenetic marks continue to be published.

Environmental toxins such as alcohol can alter the epigenome and recent evidence has supported that these epigenetic changes can be inherited across multiple generations. For investigators seeking familial disease transmissibility mechanisms beyond the genome, the inheritance of parentally acquired epigenetic change (epigenetic inheritance) has provided that alternative. For example, it has long been known that paternal alcoholism can result in deleterious effects including reduced birth weight and impaired cognitive functioning in offspring (Hegedus et al., 1984; Little and Sing, 1987). The effect of paternal alcohol exposure on two paternally methylated imprinted control regions (H19 and Rasgrf1) in paternal sperm and somatic DNA of offspring has been studied (Knezovich and Ramsay, 2012). Significant reductions in methylation at the H19 binding sites were observed in the offspring of ethanol-treated sires, and correlated with reduced postnatal weight. Interestingly, no alteration of sperm DNA methylation was observed in the offspring. The authors suggest that other epigenetic factors such as ncRNA or chromatin remodeling may be responsible for paternal transmission of the phenotype. Additionally, other toxins, such as methoxychlor, bisphenol A and the fungicide vinclozolin, have been tied to transgenerational epigenetic reprogramming and function of the male germline across generations (Skinner et al., 2013).

Much translational epigenetic study has additionally come from alcohol studies in cell populations involved in modulating stress responses. Neurons containing *Pomc* gene products, located primarily in the anterior pituitary and hypothalamus, have diverse neuroendocrine-immune and metabolic functions. These neurons have a diminished function in people with a family history of alcoholism, suggesting alcohol effects on the imprinted *Pomc* gene are transmissible across generations (Govorko et al., 2012). Alcohol-induced effects include *Pomc* hypermethylation, altered histone-modifying proteins and DNA methyltransferase levels with associated functional defects. Epigenetic modifications of *Pomc* genes are reportedly transmitted through F2 and F3 germlines, but not in female germlines.

Finally, multi-generational prenatal alcohol models have reported increased risk of gestational hyperglycemia and aberrant glucose and insulin responsiveness of offspring. The implication of an alcohol-associated hyperglycemic environment during development places subsequent generations at risk for metabolic disorders such as *diabetes mellitus*, even without subsequent fetal alcohol exposure. Specifically, a study of grandmaternal alcohol consumption in mice demonstrated transgenerational transfer of glucose intolerance (Harper et al., 2014). Sprague Dawley dams were fed ethanol liquid diets or control diets during gestational days 8–20. Dams consuming ethanol were hyperglycemic and their F1 offspring demonstrated altered glucose responsiveness, without additional alcohol exposure. A reciprocal breeding experiment using F1 Sprague–Dawley rats bred to naïve Brown Norway rats demonstrated persistent glucose intolerance in the F2 generation. This effect on glucose intolerance was normalized upon grandmaternal administration of thyroxine (T4), a thyroid hormone involved in the regulation of metabolism. This was the first experiment demonstrating that prenatal ethanol-induced alteration of glucose responsiveness can affect subsequent generations, possibly via epigenetic effects on the germ line. For all of its attractiveness as a potential mechanism of trans-generational disease, much is lacking from our understanding of epigenetic heritability-particularly in disease. As Heard and Martienssen (Heard and Martienssen, 2014) point out, much of what we may perceive as transgenerational epigenetics is confounded by the many co-factors which occur in tandem with epigenetic change and further regulate epigenetic factors. For example, there are strong correlations between differentially methylated regions and transposable elements such as LINE1 and Alu, particularly in studies of prenatal alcohol exposure (Wilhelm-Benartzi et al., 2012). Still another factor to consider is the sustainability of metabolic signatures across generations-metabolic elements which can in turn regulate the epigenetic enzymes which confer chromatin modification. These and other subtle sequence variations will make it difficult to distinguish epigenetic disease inheritance.

## CONCLUSION

The slew of transcriptional fluctuations that allow for dynamic expression to fit the specific needs of a maturing neuron are made possible only by a precise and tightly controlled regulatory system. The precision of the neurodevelopmental transcriptome is thus likely achieved by a convergence of extracellular and intrinsic cues. The work reviewed herein provides for the large and meaningful role of epigenetic mechanisms as a molecular means of such transcriptional regulation. The importance of non-coding RNA, DNA methylation, and histone modification is made even clearer by the epigenomic alteration that is demonstrated in multiple disease models. Fetal alcohol exposure has identified multiple “suspect” genes by which epigenetic dysregulation can transmit the teratogenic action of alcohol exposure. One of the most striking descriptions of epigenetic mechanisms at work in a developmental disease model was told by Kaminen-Ahola et al. (2010). The gestational exposure of mice to ethanol affected the expression of the epigenetically sensitive allele of the Agouti gene (a dominant mutation) which confers mouse coat color. Ethanol induced hypermethylation in the promoter region and increased the transcriptional repression of the gene resulting in the outwardly observable phenotype of Agouti-colored mouse coats (Kaminen-Ahola et al., 2010). This example demonstrates that fetal alcohol exposure is fully capable of translating an environmental element into molecular consequences that can result in an observable phenotype. It will be interesting to see what other environmental exposures can do to the neurodevelopmental system through epigenetic alteration. Similarly, the elucidation of such epigenetically sensitive genes in FAS and other developmental diseases is highly anticipated.

The epigenetic regulation of neurodevelopmental gene networks offers a potent and diverse mechanism for how complex neural systems are achieved. It goes without saying that characterizing every single epigenetic influence on every neurodevelopmental gene will be a long and rigorous endeavor. Epigenomic high-throughput and epigenetic editing methods perceivably will continue to quicken the pace of such findings and expand upon our current understanding of genetic–epigenetic interactions. It will certainly become more clear as the field grows that epigenetic modifications are context specific, meaning that, the changes that occur in one gene cluster or cell population do not necessarily apply to the next. This fits with our current understanding that epigenetic modifications are substantially guided by external cues often provided by the biological microenvironment. As such, it is imperative that current investigators are aware of this as they attempt to understand whole systems and tissues. Moreover, it is likely that not all epigenetic modifications are created equal. Just as specific regulatory regions govern the activity of a gene, it appears that some genomic sites are more vulnerable/receptive to epigenetic change. It will be important to isolate these sites as our focus in the epigenetic community turns from a descriptive effort to targeted modulation. Knowing that environmental-linked developmental disease is largely translated via epigenetic mechanisms, the logical progression will be investigating thresholds of epigenetic change – modifications that are necessary or sufficient to enable transcriptional change. A big hurdle to targeted epigenetic modulation will be the aforementioned fact that epigenetic modifications are widely interconnected. When these can be accurately teased out and understood, only then will we unlock the opportunity to rewrite the epigenetic codes which convey disease. Surely, the task toward elucidating gene–epigenetic relationships to the point where targeted epigenetic therapy is a possibility will be as complex as the capacities of the neural system itself.

## Conflict of Interest Statement

The authors declare that the research was conducted in the absence of any commercial or financial relationships that could be construed as a potential conflict of interest.
